# Interaction between synaptic inhibition and glial-potassium dynamics leads to diverse seizure transition modes in biophysical models of human focal seizures

**DOI:** 10.1007/s10827-016-0615-7

**Published:** 2016-08-03

**Authors:** E. C. Y. Ho, Wilson Truccolo

**Affiliations:** 1 Department of Neuroscience & Institute for Brain Science, Brown University, Providence, RI USA; 2 U.S. Department of Veterans Affairs, Center for Neurorestoration and Neurotechnology, Providence, RI USA

**Keywords:** Focal epilepsy, Seizure dynamics, Spike-wave discharges, Gamma oscillations

## Abstract

How focal seizures initiate and evolve in human neocortex remains a fundamental problem in neuroscience. Here, we use biophysical neuronal network models of neocortical patches to study how the interaction between inhibition and extracellular potassium ([*K* ^+^]_*o*_) dynamics may contribute to different types of focal seizures. Three main types of propagated focal seizures observed in recent intracortical microelectrode recordings in humans were modelled: seizures characterized by sustained (∼30−60 Hz) gamma local field potential (LFP) oscillations; seizures where the onset in the propagated site consisted of LFP spikes that later evolved into rhythmic (∼2−3 Hz) spike-wave complexes (SWCs); and seizures where a brief stage of low-amplitude fast-oscillation (∼10−20 Hz) LFPs preceded the SWC activity. Our findings are fourfold: (1) The interaction between elevated [*K* ^+^]_*o*_ (due to abnormal potassium buffering by glial cells) and the strength of synaptic inhibition plays a predominant role in shaping these three types of seizures. (2) Strengthening of inhibition leads to the onset of sustained narrowband gamma seizures. (3) Transition into SWC seizures is obtained either by the weakening of inhibitory synapses, or by a transient strengthening followed by an inhibitory breakdown (e.g. GABA depletion). This reduction or breakdown of inhibition among fast-spiking (FS) inhibitory interneurons increases their spiking activity and leads them eventually into depolarization block. Ictal spike-wave discharges in the model are then sustained solely by pyramidal neurons. (4) FS cell dynamics are also critical for seizures where the evolution into SWC activity is preceded by low-amplitude fast oscillations. Different levels of elevated [*K* ^+^]_*o*_ were important for transitions into and maintenance of sustained gamma oscillations and SWC discharges. Overall, our modelling study predicts that the interaction between inhibitory interneurons and [*K* ^+^]_*o*_ glial buffering under abnormal conditions may explain different types of ictal transitions and dynamics during propagated seizures in human focal epilepsy.

## Introduction

Epilepsy is a neurological disorder characterized by recurrent seizures affecting an estimated 65 million people worldwide (Thurman et al. 2011). Its apparent simple characterization belies the often complex and varied cellular and synaptic basis underlying the abnormal neuronal activity during epileptic seizures. As a result, despite significant advances in understanding epilepsy over the past several decades, key knowledge gaps remain with respect to the disorder. One example is the mechanism(s) concerning the transition of neural dynamics from normal to ictal as focal seizures propagate to distal regions in the brain, especially in neocortex.

Seizures have been hypothesized to originate from a wide variety of cellular and network mechanisms (Richardson et al. 2008). It is possible that, even in the same patient, different seizures might be triggered by different factors. Facing the diversity of possible triggers of seizure transition, recent theoretical studies have proposed canonical models that focus on general unifying dynamical principles (Kramer et al. 2012; Jirsa et al. 2014; Sritharan and Sarma 2014; Wang et al. 2014; Wei et al. 2014b; Naze et al. 2015). Recent animal experiments (Trevelyan et al. 2006; Zhang et al. 2012; Cammarota et al. 2013; žiburkus et al. 2013) and human studies (Truccolo et al. 2011; Schevon et al. 2012; Ahmed et al. 2014) have also suggested that complex dynamics between different types of neurons can be in play as cortical networks transition into seizures. In particular, these studies have indicated the potential critical role of fast-spiking (FS) inhibitory interneurons (see Paz and Huguenard 2015 for a review) and their interactions with changes in extracellular potassium concentration [*K* ^+^]_*o*_ (e.g. žiburkus et al. 2006). Elucidating such interplay between interneuron activity and ionic concentrations during seizure initiation and propagation can provide the much-needed clues for the development of seizure prediction, early detection and novel therapeutic approaches.

Furthermore, studies by Truccolo et al. (2011, 2014) and Wagner et al. (2015) have characterized spiking in ensembles of single neurons and local field potential (LFP) activity recorded via 96-microelectrode arrays in humans during focal seizures. Three main types of seizure activity were recorded in neocortical patches distal (∼2−4 cm) to the identified seizure onset zones: seizures consisting of sustained (∼30−60 Hz) gamma-band oscillations, seizures where the onset consisted of a direct transition into LFP spike discharges which evolved later into (∼2−3 Hz) spike-wave complexes (SWCs), and seizures where the onset included a brief transient of low voltage fast oscillations (10−20 Hz) before the evolution into SWC discharges. (For a more comprehensive classification of seizures in terms of onset patterns and relationships to pathology in larger datasets, see Perucca et al. 2014). In the gamma seizures, despite the narrowband nature of the oscillations, neuronal spiking activity remained highly irregular and asynchronous (Truccolo et al. 2014). Classification of recorded single neurons into putative principal and interneuron cells suggested that inhibition was preserved throughout the various ictal stages in these gamma seizures (Truccolo et al. 2011). On the other hand, a recent investigation of SWC seizures in these datasets indicated that inhibitory activity seems to shut off just before the emergence of large amplitude spike-wave discharges (Ahmed et al. 2014). Ahmed et al. (2014) hypothesized the role of depolarization block in interneurons and examined their sensitivity to [*K* ^+^]_*o*_ in a single cell model of a FS inhibitory interneuron.

Here, we examine how the interaction between FS inhibitory interneuron activity and extracellular potassium concentration in biophysical neuronal network models can capture the different types of propagated seizure dynamics observed in these human neocortical recordings. We used neuronal network models of neocortical patches that include pyramidal and FS interneurons (Wang and Buzsáki 1996), and [*K* ^+^]_*o*_ diffusion coupled with glial cell activity. The neuronal network models spanned 9×9 minicolumns, each 25 *μ*m wide and containing 12 pyramidal neurons and 4 FS interneurons (for a total of 9×9×16=1296 model neurons). Coupled [*K* ^+^]_*o*_ and glial buffering dynamics were adapted from previous models in Fröhlich et al. (2010).

Several previous computational models (Kager at al. 2000; Bazhenov et al. 2004; Traub et al. 2005; Anderson et al. 2007; Cressman Jr. et al. 2009; Ullah et al. 2009; Fröhlich et al. 2010; Krishnan and Bazhenov, 2011; Krishnan et al. 2013, Wei et al. 2014a, 2014b) have explored the role of intracellular and extracellular concentrations of various types of ions (e.g. potassium, sodium, chloride, calcium, etc.), as well as inhibitory/excitatory conductances and metabolic factors, on seizure activity. Our model specification was inspired by several of these previous studies, including, for example, the formulation of glial-buffering processes (see Section 2). Nevertheless, we emphasize that, to our knowledge, the work presented here is the first to examine how the interaction between inhibitory activity and abnormal levels of extracellular potassium concentration can lead to three different types of seizures (gamma, SWC, low-voltage fast-oscillations followed by SWCs) observed in intracortical recordings from patients with focal epilepsy. Overall, we demonstrate that transition into and maintenance of both gamma-band and SWC seizures in the model can be explained by variations in inhibition strength (and therefore changes in the excitation/inhibition balance) and [*K* ^+^]_*o*_ levels. We consider this to be the main novel contribution reported in our study.

## Methods

### Model description and parameter choices

#### Single neuron model

We used a single-compartment conductance based model to represent both the fast-spiking inhibitory interneurons and pyramidal cells (Wang and Buzsáki 1996; Skinner 2006; Anderson et al. 2007). The differential equation for the membrane potential of a given neuron corresponds to:

1 $$ C\times\frac{dV^{l}}{dt}=I_{int}^{l}(t)+I_{syn}^{l}(t)+I_{ext}^{l}(t) , $$

for *l*=1,2,…,1296 neurons. $I_{int}^{l}$ denotes the intrinsic current of the neuron in question, $I_{syn}^{l}$ denotes synaptic currents and $I_{ext}^{l} $denotes external input. The specific capacitance *C* is assumed to be 1 *μ*F/cm^2^. $I_{int}^{l}$ has the following general form:

2 $$\begin{array}{@{}rcl@{}} I_{int}^{l}&=&-I_{Na}-I_{K}-I_{L}-I_{KCa}\\ &=&-g_{Na}\times m_{\infty}^{3}\times h \times (V-E_{Na})\\ &&-g_{K}\times n^{4}\times(V-E_{K}([K^{+}]_{o}))\\ &&-g_{L}\times (V-E_{L}([K^{+}]_{o}))-I_{KCa}, \end{array} $$

which represent sodium, delayed-rectifier potassium, leak and calcium-mediated potassium currents, respectively. The leak reversal potential is dependent on various ionic concentrations via the Goldman-Hodgkin-Katz voltage equation:

3 $$\begin{array}{@{}rcl@{}} E_{L}({[K^{+}]_{o}})&=&26.64\text{mV}\\ &&\times\log_{e}\!\frac{1\times{[K^{+}]_{o}}\!+0.085\!\times\!{[Na^{+}]_{o}}\!+0.1\!\times{[Cl^{-}]_{i}}}{1\times{[K^{+}]_{i}}\!+ 0.085\!\times\!{[Na^{+}]_{i}}\!+0.1\!\times{[Cl^{-}]_{o}}},\\ \end{array} $$

where [*K* ^+^]_*o*_,[*K* ^+^]_*i*_,[*N* *a* ^+^]_*o*_,[*N* *a* ^+^]_*i*_,[*C* *l* ^−^]_*o*_,[*C* *l* ^−^]_*i*_ denote ionic concentrations, with subscript *i* and *o* representing intracellular and extracellular, respectively. The coefficient before each type of ionic concentration is the relative membrane permeability of that ion. The “fast” activation variable *m*_*∞*_ of the sodium current is assumed to have reached a steady state and has the form:

4 $$\begin{array}{@{}rcl@{}} m_{\infty}&=&\frac{\alpha_{m}}{\alpha_{m}+\beta_{m}},\\ \alpha_{m}(V)&=&\frac{-0.1~\text{mV}^{-1}\times(V+35~\text{mV})}{\exp(-0.1~\text{mV}^{-1}\times(V+35~\text{mV}))- 1},\\ \beta_{m}(V)&=&4\times\exp\left( -\frac{V+60~\text{mV}}{18~\text{mV}}\right), \end{array} $$

and the inactivation variable *h* of the sodium and the activation variable *n* of delayed-rectifier potassium current obey the following differential equation:

5 $$ \frac{dX}{dt}=5~\text{ms}^{-1}\times(\alpha_{X}\times(1-X)-\beta_{X}\times X), $$

where *X* can be *h* or *n*, with

6 $$\begin{array}{@{}rcl@{}} \alpha_{h}(V)&=&0.07\times\exp\left( -\frac{V+58~\text{mV}}{20~\text{mV}}\right),\\ \beta_{h}(V)&=&\frac{1}{\exp(-0.1~\text{mV}^{-1}\times(V+28~\text{mV}))+1},\\ \alpha_{n}(V)&=&-\frac{0.01~\text{mV}^{-1}\times(V+34~\text{mV})}{\exp(-0.1~\text{mV}^{-1}\times(V+34~\text{mV}))- 1},\\ \beta_{n}(V)&=&0.125\times\exp\left( -\frac{V+44~\text{mV}}{80~\text{mV}}\right). \end{array} $$

The purpose of the calcium-mediated potassium current (*I*_*KCa*_) is to provide the spike-frequency adaptation of the model pyramidal cells. It has the following form:

7 $$ I_{KCa}=g_{KCa}\times(V-E_{K})\times\frac{{[Ca^{2+}]_{i}}}{1\text{mM}+{[Ca^{2+}]_{i}}}, $$

where *g*_*KCa*_ and [*C* *a* ^2+^]_*i*_ denote the conductance for calcium-mediated potassium currents and the intracellular calcium concentration, respectively. The [*C* *a* ^2+^]_*i*_ dynamics are given according to Cressman et al. (2009) and Ullah et al. (2009):

8 $$\begin{array}{@{}rcl@{}} \frac{d{[Ca^{2+}]_{i}}}{dt}&=&-\frac{0.002\frac{\text{mol}}{\text{cm}\cdot\text{mC}}\times g_{Ca}\times(V-E_{Ca})}{1+\exp\left( -\frac{(V+25~\text{mV})}{2.5~\text{mV}}\right)}\\ &&-\frac{[Ca^{2+}]_{i}}{80~\text{ms}}, \end{array} $$

where *g*_*Ca*_ and *E*_*Ca*_ denote the conductance and reversal potential related to *C* *a* ^2+^, respectively.

#### Network structure and connectivity

**Fig. 1:**
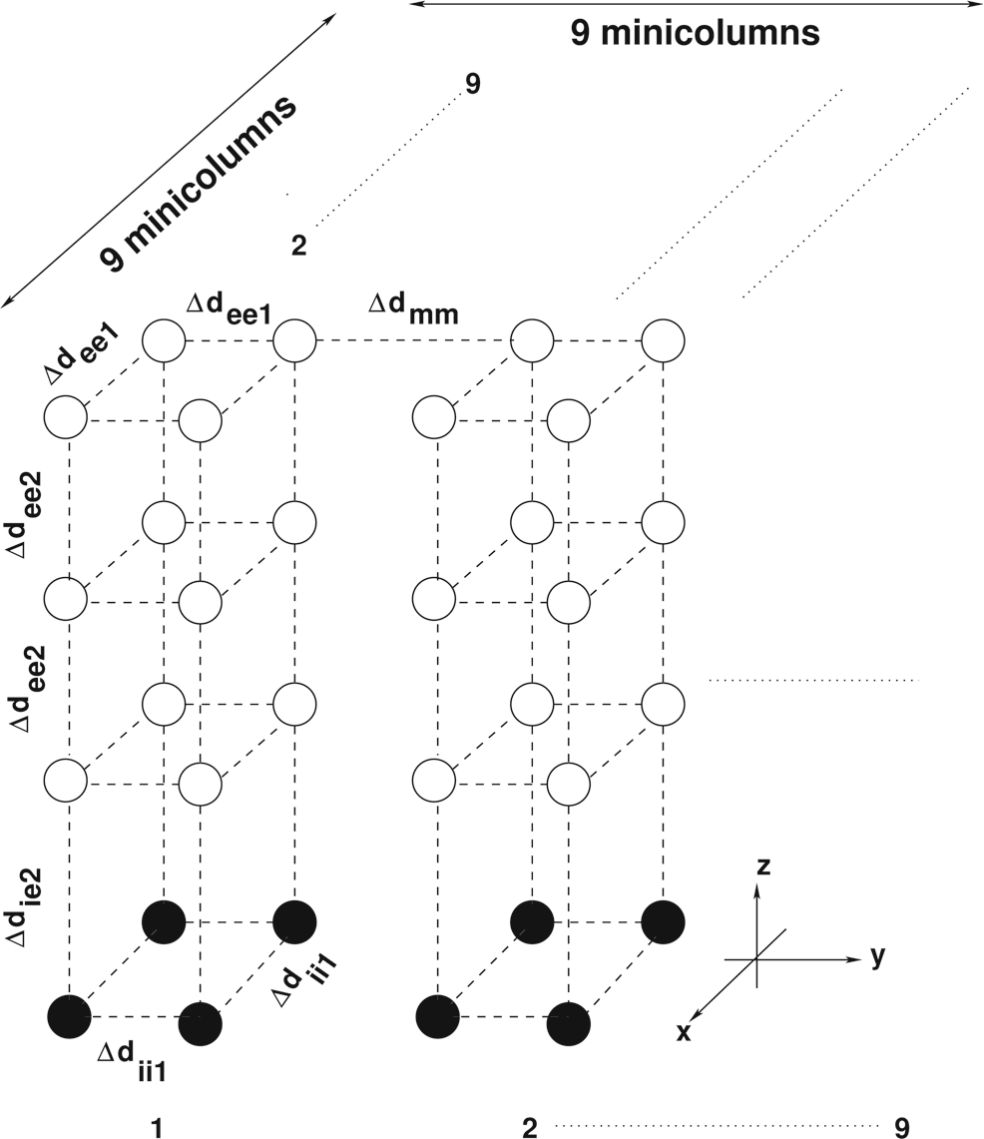
Model cortical network: spatial configuration. Open circles represent pyramidal cells and black circles represent inhibitory interneurons. Two minicolumns are shown. Table 1 specifies the values for the distance parameters in the figure

The structure of our network of model neurons is based on the arrangement of neurons in minicolumns in the neocortical area (Buxhoeveden and Casanova 2002). In this network model, each minicolumn consists of 16 single-compartment model neurons, of which 12 are excitatory pyramidal cells and 4 are inhibitory interneurons. The neurons are arranged into four layers within each minicolumn (Fig. 1). Each layer consists of four neurons in a square arrangement, with each vertex of the square being the location of one neuron. Pyramidal cells occupy the top three layer and the bottom layer is for the four inhibitory interneurons. (We do not attempt here to replicate the actual 6-layer neocortical structure). The entire cortical network consists of 9 ×9 such minicolumns for a total of 1296 model neurons. Details of the structure configuration of the cortical network model and various distance parameters between model neurons are given in Fig. 1 and Table 1. Unless otherwise stated, we chose a global random connectivity to avoid boundary effects associated with the small size of the simulated cortical network.

**Table 1:** Network geometry parameters. Please refer to Fig. 1 for the description of parameters

Parameter	Units	Values
Δ*d*_*ee*1_	cm	5×10^−4^
Δ*d*_*ee*2_	cm	5×10^−4^
Δ*d*_*ie*2_	cm	30×10^−4^
Δ*d*_*ii*1_	cm	5×10^−4^
Δ*d*_*mm*_	cm	15×10^−4^

#### Glial and potassium ionic dynamics

Each neuron in the network is endowed with a potassium pump. An associated potassium uptake dynamics related to nearby glial cells are included in the model to regulate the extracellular potassium concentration [*K* ^+^]_*o*_. The potassium pump is represented by:

9 $$ {I_{Kpump}}=\frac{{I_{Kmax}}}{\left( 1+\frac{{[K^{+}]_{o(eq)}}}{{[K^{+}]_{o}}}\right)^{2}}, $$

where *I*_*Kmax*_ and [*K* ^+^]_*o*(*eq*)_ are defined in Table 3. The glial potassium uptake mechanism is modelled in terms of a reversible binding process between extracellular potassium ions and the glial buffer (Kager et al. 2000; Bazhenov et al. 2004; Park and Durand 2006; Fröhlich et al. 2008, 2010). The binding/unbinding process can be represented as

10 $$K^{+}+B\rightleftharpoons[{k_{b}}]{{k_{f}}} KB, $$

where *K* ^+^ and *B* denote the extracellular potassium ions and the free buffer in the glia, respectively, and *k*_*b*_ and *k*_*f*_ correspond to the backward and forward glial binding rate, respectively (Tables 3 and 4). *K* ^+^ and *B* combine reversibly to form the bound form of the buffer *KB*. We fixed the maximal amount of free glial buffer [*B*]_*max*_ to 500mM (Table 3, see also Kager et al. 2000).

The above process (10) can be written as a set of differential equations:

11 $$\begin{array}{@{}rcl@{}} {{\frac{\partial {[K^{+}]_{o}}}{\partial t}|}_{\text{glia}}}\!&=&\!{k_{b}}\!\times\!\frac{({[B]_{max}}-{[B]})}{1.1}-{k_{f}} \times{[K^{+}]_{o}}\times{[B]},\\ \frac{d{[B]}}{dt}&=&{k_{b}}\times({[B]_{max}}-{[B]})-{k_{f}}\!\times{[K^{+}]_{o}}\times{[B]},\\ {k_{f}}&=& \frac{{k_{b}}/1\text{mM}}{1+\exp\left( \frac{{[K^{+}]_{o}}-{[K^{+}]_{o(th)}}}{\theta}\right)}, \end{array} $$

where [*K* ^+^]_*o*(*th*)_ and *𝜃* are specified in Table 3.

We vary [*K* ^+^]_*o*(*th*)_and *𝜃* to simulate a range of physiological and pathological states for the potassium glial-buffering system. [*K* ^+^]_*o*(*th*)_ corresponds to the equilibrium level (set point) for the extracellular potassium concentration being targeted by the glial buffering system. How tightly the glial buffering system tracks this equilibrium set-point depends on the parameter *𝜃*. For example, the lower the absolute values of *𝜃*, the closer the glial buffer system attempts to keep the [*K* ^+^]_*o*_ concentration to the target [*K* ^+^]_*o*(*th*)_ level. Under this condition, the system may fail if there is a big enough difference between [*K* ^+^]_*o*_ and [*K* ^+^]_*o*(*th*)_, leading to bi-stability and abnormal functioning. In more physiological conditions, the absolute value of *𝜃* is kept at higher values so that the glial buffering system tracks [*K* ^+^]_*o*(*th*)_ in a softer fashion, allowing a broader range of [*K* ^+^]_*o*_ levels and more effectively avoiding bi-stability. We note that while we vary [*K* ^+^]_*o*(*th*)_ and *𝜃* in order to change the glial buffering dynamics and to induce abnormal states, we do not yet have a hypothesis about specific causes that would lead to these changes in first place.

The diffusion of extracellular potassium ions across the network was modelled according to the diffusion equation:

12 $$ \left.\frac{\partial [K^{+}]_{o}}{\partial t}\right|_{\text{diff}}=D\times\nabla^{2}{[K^{+}]_{o}}, $$

where ∇^2^ is the Laplace operator $\frac {\partial ^{2}}{\partial x^{2}}+\frac {\partial ^{2}}{\partial y^{2}}+\frac {\partial ^{2}}{\partial z^{2}}$ and *D* is the diffusion constant. In the simulations, ∇^2^ is approximated by a set of finite difference equations, since the model neurons are discretely located (Fig. 1). We impose the “no-flux” or Neumann boundary condition (i.e. $\frac {\partial {{[K^{+}]_{o}}}}{\partial x}=0$ on the *y*−*z* planes at both the *x* boundaries and so on) in the network simulations.

Therefore, the total time derivative of [*K* ^+^]_*o*_ of a particular model neuron, $\frac {d{[K^{+}]_{o}}}{dt}$ (unit in mM ⋅*ms* ^−1^), is the sum of all the potassium, pump, glia and diffusion currents

13 $$\begin{array}{@{}rcl@{}} \frac{d{[K^{+}]_{o}}}{dt}&=&\frac{50}{96489}\frac{\text{mol}}{\text{cm}\cdot\text{mC}}\times(I_{KCa}+I_{K}- I_{Kpump}\\ &&+g_{L}|_{[K^{+}]}\times(V-E_{K}({[K^{+}]_{o}})))\\ &&+ \left.\frac{\partial {[K^{+}]_{o}}}{\partial t}\right|_{\text{glia}}+ \left.\frac{\partial [K^{+}]_{o}}{\partial t}\right|_{\text{diff}}, \end{array} $$

where $g_{L}|_{[K^{+}]}$ (≥0) is the value of the leak conductance attributable to potassium. The value can be estimated by fitting the equation,

$$\begin{array}{@{}rcl@{}} g_{L}\times(V-E_{L}({[K^{+}]_{o}}))&=&g_{L}|_{[K^{+}]}\times(V-E_{K}({[K^{+}]_{o}}))\\ &&+g_{L}|_{[Cl^{-}]} \times(V-E_{Cl})\\ &&+ g_{L}|_{[Na^{+}]}\times(V-E_{Na}), \end{array} $$

with the constraint that

14 $$ g_{L}=g_{L}|_{[K^{+}]}+g_{L}|_{[Cl^{-}]}+g_{L}|_{[Na^{+}]}, $$

where $g_{L}|_{[Cl^{-}]}$ (≥0) and $g_{L}|_{[Na^{+}]}$ (≥0) are the values of leak conductance due to *C* *l* ^−^ and *N* *a* ^+^ respectively, while *E*_*Cl*_ and *E*_*Na*_ are their respective reversal potentials as shown in equations (16) and (17). $g_{L}|_{[K^{+}]}$ values at ∼ 6–13 % of total leak give reasonable fits over a wide range of physiological [*K* ^+^]_*o*_ and membrane potential values. (Various fitting scenarios indicate that >70 % of the total leak conductance value is due to chloride). Based on the above procedure, we assume a $g_{L}|_{[K^{+}]}$ value at 6 % of the *g*_*L*_ value.

The potassium reversal potential *E*_*K*_ is affected by [*K* ^+^]_*o*_ via the Nernst equation:

15 $$ E_{K}=26.64\text{mV}\times\log_{e}\left( \frac{{[K^{+}]_{o}}}{{[K^{+}]_{i}}}\right), $$

where the intracellular potassium concentration [*K* ^+^]_*i*_ is set at 133 mM (Table 3). Similarly, *E*_*Cl*_ is given by

16 $$ E_{Cl}=26.64\text{mV}\times\log_{e}\left( \frac{{[Cl^{-}]_{i}}}{{[Cl^{-}]_{o}}}\right)\sim-74\text{mV}, $$

and *E*_*Na*_ by

17 $$ E_{Na}=26.64\text{mV}\times\log_{e}\left( \frac{{[Na^{+}]_{o}}}{{[Na^{+}]_{i}}}\right)\sim54\text{mV}. $$

See also Table 3 for values of [*N* *a* ^+^]_*o*_, [*N* *a* ^+^]_*i*_, [*C* *l* ^−^]_*o*_ and [*C* *l* ^−^]_*i*_.

#### Synaptic model

Unless otherwise stated, the synaptic current ${I_{syn}^{l}}$ corresponds to

$$\begin{array}{@{}rcl@{}} {I_{syn}^{l}}&=&\sum\limits_{m\ne l}\Gamma_{{m}\rightarrow{l}}\times {g_{syn}^{m\rightarrow l}}\times s_{m}(t\,-\,\tau_{delay})\times(E_{\{e,i\}}\,-\,V^{l}), \end{array} $$

with *s*_⋅_(*t*) representing the synaptic gating variable of each model neuron, such that

18 $$\begin{array}{@{}rcl@{}} s_{l}&\rightarrow& s_{l}+{s_{max}}\;(\text{when }V^{l}\text{ crosses 0 from below}),\\ \frac{ds_{l}}{dt}&=&-\frac{s_{l}}{{\tau_{\{e,i\}}}}\;\text{(otherwise)}. \end{array} $$

In the above, Γ_*m*→*l*_ denotes the binary connectivity matrix element in which a value of 1 represents the existence of a synaptic connection in neuron *l* from neuron *m*, and a value 0 represents the absence of such connection. Table 5 lists the probabilities of connection between pyramidal neurons (*P*(*e*→*e*)), from pyramidal neurons to interneurons (*P*(*e*→*i*)), from interneurons to pyramidal neurons (*P*(*i*→*e*)) and between interneurons (*P*(*i*→*i*)). The term *E*_{*e*,*i*}_ denotes the excitatory or inhibitory reversal potential value, respectively. (Whether the reversal potential is excitatory or inhibitory depends on the particular neuron–i.e. neuron *m*–from which neuron *l* receives synaptic current).

As done in Ho et al. (2012) for computational efficiency, we use a discontinuous model to represent the opening of the synapses (equation set (18)) whenever there is a spike. Parameters *τ*_{*e*,*i*}_ and *s*_*max*_ (for *s*_*l*_) denote the excitatory (inhibitory) synaptic decay time constant and the value of the maximal opening of the synaptic gates per spike, respectively (whether the decay time constant should be excitatory or inhibitory depends on whether neuron *l* itself is a pyramidal cell or an inhibitory interneuron). Table 5 provides more details on the terms of the above equations.

#### Background synaptic activity

Unless otherwise stated, each model neuron is driven by an external input *I*_*ext*_ with the form (Rudolph et al. 2004) to mimic the activities of the fluctuating synaptic background:

19 $$\begin{array}{@{}rcl@{}} I_{ext}^{l}\!&=&\!g_{e}^{l}(t)\times({E_{e}}-V^{l})+g_{i}^{l}(t)\times(E_{i}-V^{l}),\\ \frac{d{g^{\;\;l}_{\{e,i\}}}(t)}{dt}\!&=&\!\frac{{g^{}_{\{e,i\}0}}\,-\,{g^{\;\;l}_{\{e,i\}}}(t)}{{\tau_{\{e,i\}}}}+\! \sqrt{\frac{2\times{\sigma^{2}_{\{e,i\}}}}{{\tau_{\{e,i\}}}}}\!\times\!\chi_{\{e,i\}}^{\;\;l}(t) ,\\ \end{array} $$

where *E*_{*e*,*i*}_ denotes the excitatory/inhibitory reversal potential and *τ*_{*e*,*i*}_ is the excitatory/inhibitory time constant. The excitatory/inhibitory conductance values *g*_{*e*,*i*}_ are stochastic variables following an Ornstein-Uhlenbeck process (equation set (19)), with mean *g*_{*e*,*i*}0_ and SD *σ*_{*e*,*i*}_ (excitatory/inhibitory fluctuations). The stochastic term *χ*_{*e*,*i*}_ is such that ${\int }_{0}^{\epsilon }\chi _{\{e,i\}}(s)\times ds$ is a Gaussian distributed random variable with mean zero and variance *𝜖*>0. Further details on the numerical implementation of the stochastic elements of equation set (19) can be found in Ho et al. (2012).

Table 5 provides more details on the terms in the above equation.

### Implementing the simulations

**Fig. 2:**
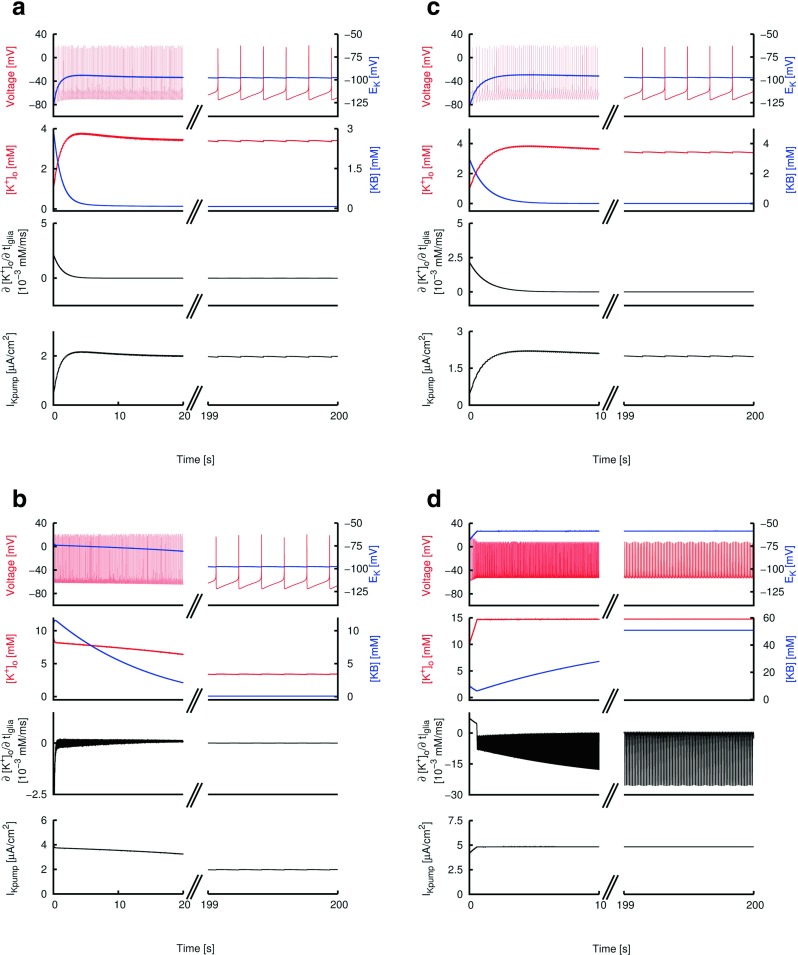
Four single neuron model simulations with physiological and abnormal glial parameters. Bi-stability is observed with abnormal glial parameters. In each of **a**, **b**, **c** and **d**: Top panel shows the membrane potential (*red*) and potassium reversal potential *E*_*K*_ (*blue*). Second panel shows the extracellular potassium concentration [*K* ^+^]_*o*_ (*red*) and the glial bound buffer concentration [*K* *B*] (*blue*). Third panel shows the rate of change of extracellular potassium concentration due to the glial action ${{\frac {\partial [K^{+}]_{o}}{\partial t}|}_{\text {glia}}}$. Bottom panel shows the potassium pump current *I*_*Kpump*_. **(a)**“Physiological” parameters. Initial conditions [*K* ^+^]_*o*_(*t*=0)=1mM, [*K* *B*](*t*=0)=3mM. **(b)**“Physiological” parameters. Initial conditions [*K* ^+^]_*o*_(*t*=0)=10mM, [*K* *B*](*t*=0)=10mM. **(c)**“Pathological” parameters. Initial conditions [*K* ^+^]_*o*_(*t*=0)=1mM, [*K* *B*](*t*=0)=3mM. **(d)**“Pathological” parameters. Initial conditions [*K* ^+^]_*o*_(*t*=0)=10mM, [*K* *B*](*t*=0)=10mM

The code for the model cortical network simulation was written in C++ (gcc, version 4.4.7, http://gcc.gnu.org). We used open MPI (version 1.8, http://www.open-mpi.org) for parallel processing of each simulation. Each typical 1296-neuron simulation used eight CPUs. A 10-hour simulation on the Brown University computer cluster (https://www.ccv.brown.edu/) usually yielded ∼ 2-3 minutes of data. Parameter scanning was automated via the use of custom Perl (version 5.18.4) and Linux shell scripts. Post simulation analysis of data was carried out using Matlab (version R2013b, http://www.mathworks.com), Octave (version 3.8.1, http://www.gnu.org/software/octave/) and customized Perl scripts. Single neuron simulations (Fig. 2) were performed using xppaut (Ermentrout 2002). Graphical rendering of data was performed by gnuplot (version 4.6.3, http://www.gnuplot.info), xfig (version 3.2) and gimp (version 2.8.10, http://www.gimp.org). The parula palette of the time-frequency plots is from https://github.com/Gnuplotting/gnuplot-palettes. The C++ code is available in the NEURON ModelDB database (https://senselab.med.yale.edu/ModelDB/showModel.cshtml?model=190306). The ModelDB accession number for the network model reported in this paper is 190306.

## Results

We build model neocortical networks (Fig. 1) using conductance-based model neurons endowed with glial dynamics for potassium uptake. Based on the observations of human focal seizure data (LFPs and spiking activity in ensembles of single neurons), our aim is to use these model networks to reproduce the neural dynamics during the transition into ictal states of propagated focal seizures. Our presentation is structured as follows. First, based on both single neuron and network simulations (Section 2), we demonstrate that pathological glial uptake of potassium can lead to bi-stable dynamics in the model cortical network. The bi-stable dynamics consist of a “high-activity” state characterized by an elevated [*K* ^+^]_*o*_ level, and a state of “low-activity” with a low [*K* ^+^]_*o*_ level. We relate the “high-activity” state to “ictal” events and the “low-activity” state to non-epileptic, normal activity. Although network bistability has been implicated in computational studies as an avenue for seizure transition (Fröhlich et al. 2010), here we demonstrate that abnormal glial buffering can increase the propensity of single neurons (and thus the cortical network) to exhibit bi-stability (Fig. 2). The underlying mechanism for the bistabilty is the imbalance in the reversible reaction of the potassium buffer between the bound and unbound forms (10).

Second, with the seizure transition mechanism in place for the model cortical network, we then examine how certain types of synaptic changes involving mostly inhibitory interneurons and their interaction with potassium extracellular concentration may lead to various types of seizure transitions as observed in the human data. Our strategy is to explore inhibitory synaptic and intrinsic parameters relevant to the occurrence of depolarization block in model inhibitory interneurons. We also note that, in our model, pyramidal neurons are not as prone to depolarization block (in comparison to inhibitory interneurons) because of their *C* *a* ^2+^-mediated potassium conductances (*g*_*KCa*_) and the corresponding after-spike hyperpolarization effects (7).

**Fig. 3:**
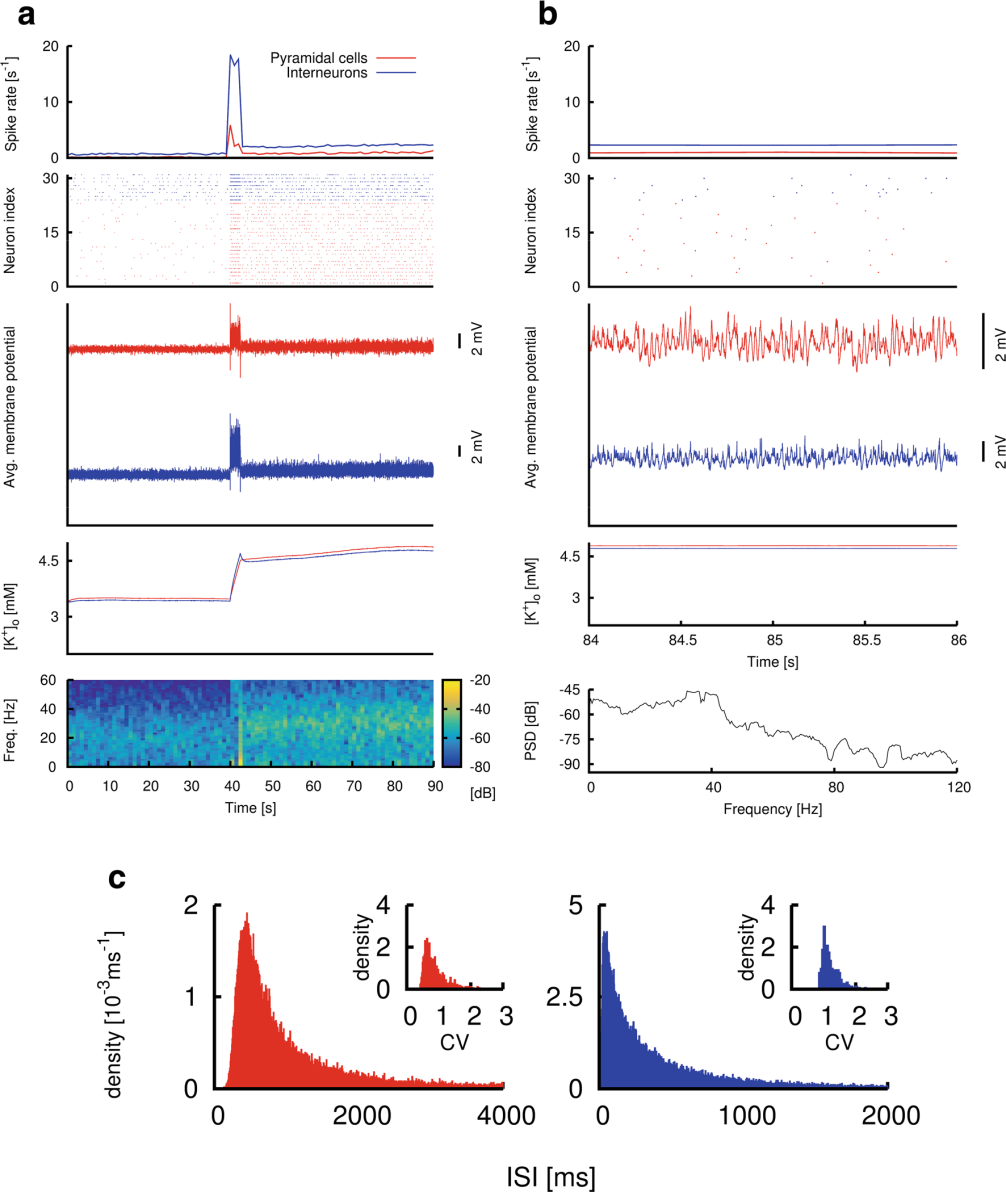
An example gamma seizure simulation with pathological glial and pump parameters. **(a)** Depiction of neural activity and time course of various biophysical parameters during a 90-second simulation. The network starts with a “low-activity” non-seizure state (first 40 seconds of simulation). A DC stimulation is applied to every neuron in the network between 40 and 42.5 seconds, after which the system is “kick-started” into a high activity state with an elevated level of extracellular potassium (after 42.5 seconds). **Topmost panel:** average spike rate of pyramidal cells (*red*) and interneurons (blue) during the simulation. **Second panel:** raster plot of action potentials of neurons in 2 of the 81 simulated minicolumns. These 2 minicolumns are located in the centre of the network. **Third panel:** average membrane potential values of interneurons (*blue*) and pyramidal cells (*red*) during simulation. **Fourth panel:** average extracellular potassium level values for interneurons (blue) and pyramidal cells (*red*) during simulation. **Fifth panel:** Time-frequency plot of the average membrane potential values of the pyramidal cells (third panel-red). Clear sustained gamma band oscillations (∼40 Hz) at the population level emerge with the DC stimulation and persist even after the stimulation is terminated. **(b)** A 2-second segment from the 90-second simulation in **a**. Same conventions as in **a**, except that the bottom panel is a power spectral density (PSD) plot of the 2-second average membrane potential values of the pyramidal cells. The maximum of the PSD is located in the gamma range. **(c)** Histograms of interspike intervals (ISIs) of all the pyramidal cells (red) and interneurons (blue) during gamma population activity ($t \gtrsim 45$ seconds). **Insets:** Histograms of the ISI coefficients of variation (CV) for spike trains of individual pyramidal cells (red) and interneurons (blue), also during gamma population activity ($t \gtrsim 45$ seconds)

**Fig. 4:**
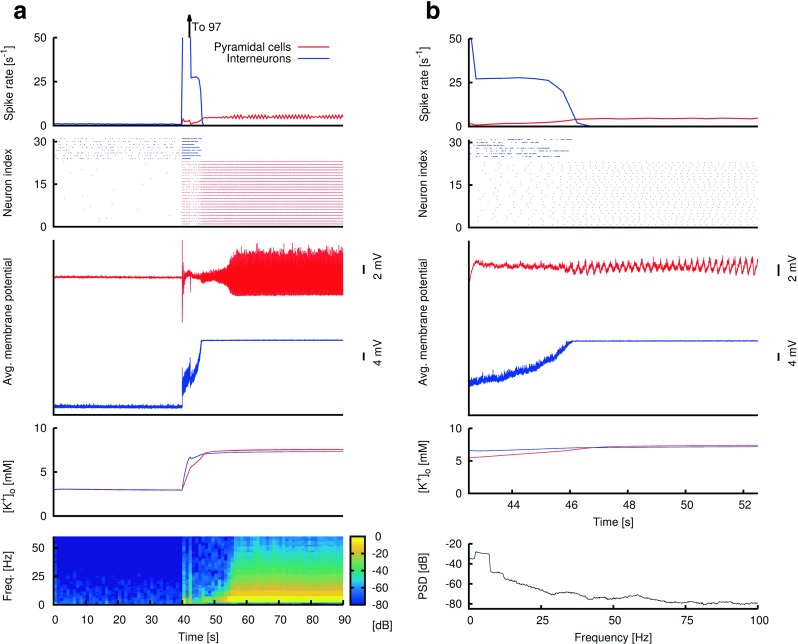
An example simulation showing a transition into a spike-wave-complex (SWC) seizure via low inhibitory synaptic strength. **(a)** Depiction of a 90-second simulation showing several biophysical variables. As in Fig. 3, the network starts with a “low-activity” non-seizure state (first 40 seconds of simulation). DC stimulation is applied to every neuron between 40 and 42.5 seconds. **Topmost panel:** average spike rate of pyramidal cells (*red*) and interneurons (*blue*) during the simulation. As rhythmic SWC discharges emerge, the firing rate of pyramidal neurons approaches ∼5 Hz. **Second panel from top:** raster plot of action potentials for neurons in 2 of the simulated 81 minicolumns. The location of these 2 minicolumns is the same as in Fig. 3. **Third panel:** average membrane potential values of interneurons (blue) and pyramidal cells (*red*) during simulation. **Fourth panel:** average extracellular potassium level values for interneurons (*blue*) and pyramidal cells (red). **Fifth panel:** Time-frequency plot of the average membrane potential values for the pyramidal neurons (third panel-red). The power peak at ∼5 Hz (after the DC stimulation) corresponds to the emergence and maintenance of rhythmic SWC discharges, even after the DC stimulation is terminated. **(b)** A 10-second selection from the 90-second simulation in (a) showing the transition (after the DC stimulation) into SWCs. Same convention as in (a), except the bottom panel is a power spectral density (PSD) plot of the 10-second average membrane potential values of the pyramidal cells (third panel-red). The cessation of spiking of interneurons at around *t*=46 seconds (first and second panels) as they enter depolarization block (third panel-blue) is concomitant with the emergence of SWCs of increasing amplitude (third panel-red)

**Fig. 5:**
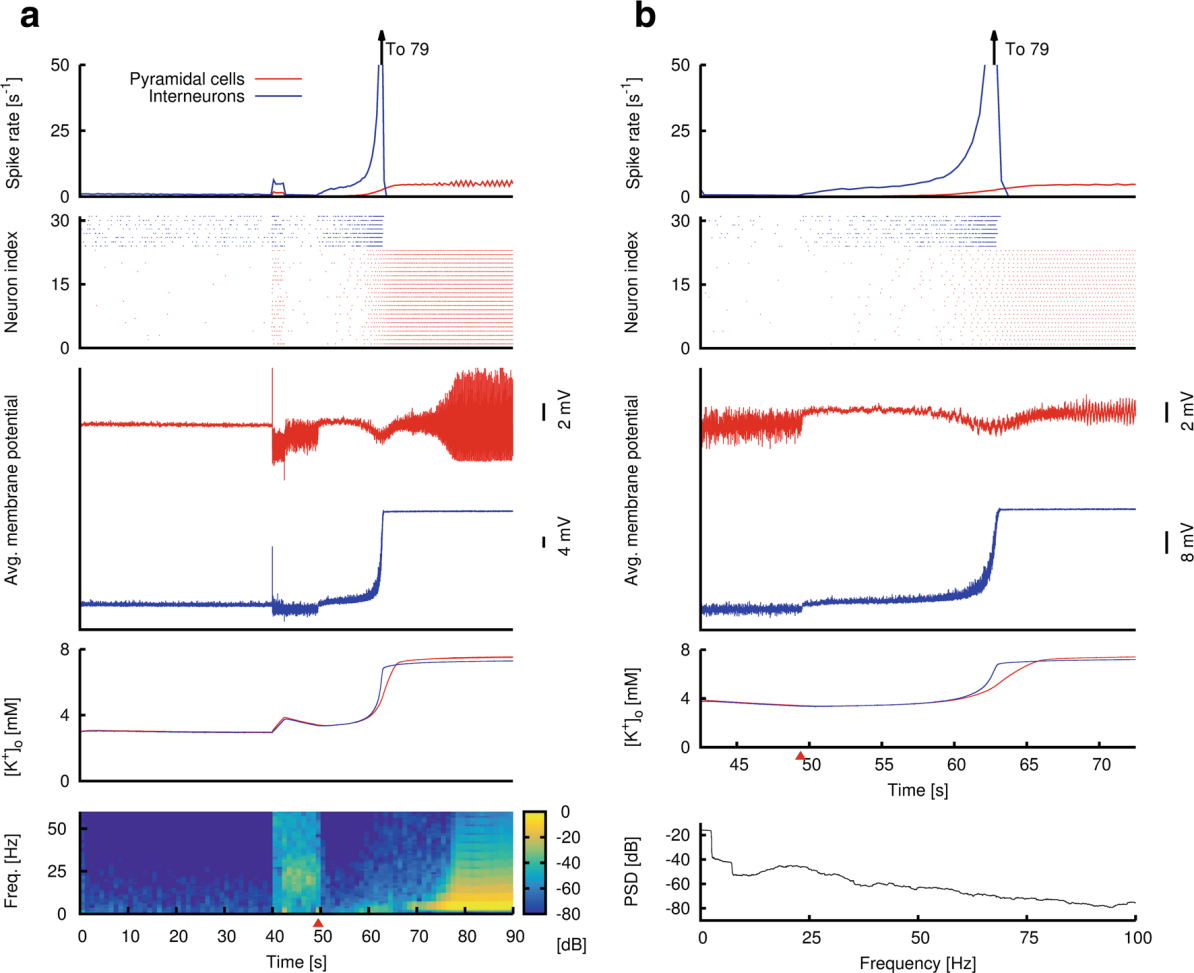
An example simulation showing transition into low voltage fast (∼20 Hz) oscillation followed by SWC discharges, obtained via initial high inhibitory synaptic strength and subsequent depletion of inhibition. **(a)** Depiction of a 90-second simulation showing several biophysical variables. The network spends its first 40 seconds in a low-activity “non-epileptic” state. A DC stimulation is applied to every neuron in the network between 40 and 42.5 seconds. During this DC stimulation period, the effective inhibition of the network is also drastically increased to mimic the build up of inhibitory strength prior to seizure (unlike in Figs. 3 and 4 where the effective inhibition is kept constant throughout the entire simulation). This increased effective inhibition is held constant up to *t*=49.5 seconds, after which inhibition wears off to zero (starting point of this depletion phase is marked by the red triangle). **Topmost panel:** average spike rate of pyramidal cells (*red*) and interneurons (*blue*) during the simulation. **Second panel:** raster plot of action potentials of neurons in 2 of the 81 minicolumns simulated. The location of these 2 minicolumns is the same as in Figs. 3 and 4. **Third panel:** average membrane potential values of interneurons (*blue*) and pyramidal cells (*red*). **Fourth panel:** average extracellular potassium level values for interneurons (*blue*) and pyramidal cells (*red*). **Fifth panel:** time-frequency plot of the average membrane potential values of the pyramidal cells (third panel). **(b)** A 30-second selection from the 90-second simulation in **a** showing both the transient low voltage fast (∼20 Hz) oscillations and the subsequent emergence of rhythmic SWC discharges. Same convention as in **a**, except the bottom panel is a power spectral density (PSD) plot of the 30-second average membrane potential values of the pyramidal cells. Transient (∼ 20 Hz) oscillations are observed after the DC stimulation but before inhibition wears off starting at *t*=49.5 seconds (red triangle). As inhibition begins to wear off, the firing rate of interneurons shows an initial transient increase, after which it decreases to zero when the interneurons enter depolarization block (third panel-blue, after around *t*=50 seconds). The reduced inhibition (due to both active wearing off of inhibition and the cessation of interneuron spiking) and increased [*K* ^+^]_*o*_ (fourth panel) also lead to the increased firing of pyramidal cells and the emergence of rhythmic SWC discharges (third panel-red, towards the end)

Variations of parameters related to synaptic inhibition and glial potassium buffering allow us to replicate in detail three types of seizure patterns observed in the human data (gamma seizures and two types of spike-and-wave seizures; Truccolo et al. 2014). Thus, all three types of seizures can each be linked, via the same computational network model, to specific temporal patterns of synaptic activities and glial buffering dynamics during the evolution of propagated ictal states (Figs. 3, 4 and 5). Table 2 summarizes the results of our network simulations in terms of the paths leading to the three types of seizure transitions. All of the primary variables and parameters used in the simulations and analyses below are introduced and defined in Tables 1, 3, 4 and 5.

**Table 2:** Pathways to three main seizure types in the focal seizure model

Seizure type	Synaptic inhibition	Interneuron depolarization block	Representative simulation
Gamma	Strong synaptic inhibition	No	Fig. 3
Spike-wave complex (SWC)	Weak synaptic inhibition	Yes	Fig. 4
Low-voltage fast oscillations followed by SWCs	Strong synaptic inhibition at seizure onset, followed by a weakening of inhibition (e.g. GABA depletion).	Yes	Fig. 5

**Table 3:** Intrinsic, glial and ionic parameters used for network simulations

Parameter	Description	Units	Values	
			Pyramidal	Interneurons
*C*	Specific neuron membrane capacitance	*μ*F/cm^2^	1	1
*g*_*Na*_	Sodium conductance	mS/cm^2^	35	35
*g*_*K*_	Potassium conductance	mS/cm^2^	9	S
*g*_*L*_	Leak conductance	mS/cm^2^	0.135	0.1
*g*_*KCa*_	Ca ^2+^-activated potassium conductance	mS/cm^2^	S	0
*g*_*Ca*_	Calcium conductance	mS/cm^2^	0.1	0
*E*_*Na*_	Sodium reversal potential	mV	54	54
*E*_*Ca*_	Calcium reversal potential	mV	120	120
*I*_*ext*_	External applied current to model neurons	*μ*A/cm^2^	S	S
[*K* ^+^]_*i*_	Intracellular potassium concentration	mM	133	133
[*N* *a* ^+^]_*o*_	Extracellular sodium concentration	mM	130	130
[*N* *a* ^+^]_*i*_	Intracellular sodium concentration	mM	17	17
[*C* *l* ^−^]_*o*_	Extracellular chloride concentration	mM	130	130
[*C* *l* ^−^]_*i*_	Intracellular chloride concentration	mM	8	8
*D*	Diffusion constant of *K* ^+^ in extracellular space (eqn. (12))	cm ^2^/ms	2.5×10^−9^	2.5×10^−9^
*I*_*Kmax*_	Maximal potassium pump current	*μ*A/cm^2^	S	S
[*K* ^+^]_*o*(*eq*)_	Equilibrium extracellular potassium concentration of *I*_*Kpump*_	mM	S	S
[*B*]_*max*_	Maximal glial free buffer concentration (eqn. set (11))	mM	500	500
*k*_*b*_	Backward glial unbinding rate (eqn. set (11))	ms ^−1^	0.0008	0.0008
[*K* ^+^]_*o*(*th*)_	Threshold extracellular potassium concentration of glial buffer (eqn. set (11))	mM	S	S
*𝜃*	see eqn. set (11)	mM	S	S

An “S” on the value column denotes that the parameter is dependent on specific simulations. Please refer to the main text for specific values

**Table 4:** Variables for network simulations

Variable	Description (one variable for each modeled neuron)	Units
[*K* ^+^]_*o*_	Extracellular potassium concentration (eqn. (13))	mM
*E*_*K*_	Potassium reversal potential (eqn. (15))	mV
*E*_*L*_	Leak reversal potential (eqn. (3))	mV
[*C* *a* ^2+^]_*i*_	Intracellular calcium concentration (eqn. (8))	mM
[*B*]	Glial free buffer concentration (eqn. set (11))	mM
[*K* *B*]	Glial bound buffer concentration ([*K* *B*]≡[*B*]_*max*_−[*B*])	mM
*k*_*f*_	Forward glial binding rate (eqn. sets (10) and (11))	ms ^−1^⋅mM ^−1^
*s*	Synaptic variable of each neuron (eqn. set (18))	-
*g*_*e*_	Background excitatory conductance (eqn. set (19))	mS/cm ^2^
*g*_*i*_	Background inhibitory conductance (eqn. set (19))	mS/cm ^2^
*V*	Membrane potential	mV
*h*	Sodium current inactivation variable	-
*n*	Potassium current activation variable	-
$m_{\infty }$	Sodium current fast activation variable	-
*t*	Time (independent variable)	ms
*x*,*y*,*z*	Spatial coordinates (independent variables eqn. set (12))	cm

**Table 5:** Synaptic parameters used for network simulations

Parameter	Description	Units	Value
*E*_*i*_	Inhibitory reversal potential	mV	-72
*E*_*e*_	Excitatory reversal potential	mV	0
*τ*_*i*_	Inhibitory synaptic decay time constant	ms	5
*τ*_*e*_	Excitatory synaptic decay time constant	ms	3
*τ*_*delay*_	Axonal conduction delay	ms	0.5
*s*_*max*_	Maximal value of gating variable per spike	-	1, except for Fig. 5 where it varies in time for interneurons
$s_{max}^{term}$	Terminal value of maximal gating variable per spike (Figure 5, interneurons only–equation (21))	-	70
*τ*_*rise*_	see Eq. (21). Figure 5; interneurons only.	ms	62.5
*g*_*e*0_	Mean background excitatory drive to model neurons	mS/cm ^2^	S
*σ*_*e*_	Background excitatory fluctuation level	mS/cm^2^	S
*g*_*i*0_	Mean background inhibitory drive to model neurons	mS/cm ^2^	S
*σ*_*i*_	Background inhibitory fluctuation level	mS/cm^2^	S
${g_{syn}^{{i}\rightarrow {i}}}$	Unitary conductance from an inhibitory interneuron to another interneuron	mS/cm^2^	S
${g_{syn}^{{e}\rightarrow {e}}}$	Unitary conductance from a pyramidal cell to another pyramidal cell	mS/cm^2^	S
${g_{syn}^{{e}\rightarrow {i}}}$	Unitary conductance from a pyramidal cell to another interneuron	mS/cm^2^	S
${g_{syn}^{{i}\rightarrow {e}}}$	Unitary conductance from an interneuron to another pyramidal cell	mS/cm^2^	S
$P(i\rightarrow i)$	Connection probability from one interneuron to another interneuron	-	0.25
$P(e\rightarrow e)$	Connection probability from one pyramidal cell to another pyramidal cell	-	0.15
$P(e\rightarrow i)$	Connection probability from one pyramidal cell to another interneuron	-	0.15
$P(i\rightarrow e)$	Connection probability from one interneuron to another pyramidal cell	-	0.25
$\Gamma _{{m}\rightarrow {n}}$	Connectivity matrix element (with values of either 1 or 0) between neuron *m* and *n*	-	assigned according to the four probability values above

An “S” on the value column denotes that the parameter is dependent on a specific simulation. Please refer to the main text for specific values

### Firing properties of the single-neuron models under physiological and abnormal glia uptake conditions

We used four single pyramidal cell model simulations to show the effects of the potassium pump and glial dynamics on neuronal excitability (Fig. 2). In each of these four simulations, *I*_*ext*_=0.36, [*K* ^+^]_*o*(*eq*)_=3, [*K* ^+^]_*o*(*th*)_=15, *I*_*Kmax*_=7, *g*_*KCa*_=5. In this section, we define the “physiological” value *𝜃*=−1.15, and the “pathological” value as *𝜃*=−0.05 (equation set (11)). Table 3 specifies the units of these parameters. Our aim is to show that the neural system tends to settle into a single steady state for a wide range of initial conditions when the increase in the forward binding rate *k*_*f*_ is smooth with increasing [*K* ^+^]_*o*_ (as in the physiological case, effected by a larger absolute value of *𝜃* – see equation set (11)).

However, when there is a sharper increase in *k*_*f*_ with increasing [*K* ^+^]_*o*_ (as in the pathological case, effected by a decreased absolute value of *𝜃*), some of the initial conditions can lead the neural system into a state where virtually all of the glial buffers are free (i.e. [*K* *B*]→0). In this case, the system’s ability to return potassium to the extracellular space and maintain a target value of extracellular potassium level is impaired. The impairment occurs because Eq. (10) is no longer reversible when [*K* *B*]→0.

In Fig. 2a and b, we show two single pyramidal neuron model simulations with the same set of “physiological” glial and potassium pump parameters but with different [*K* ^+^]_*o*_ and [*B*] initial conditions. In Fig. 2a, we set [*K* ^+^]_*o*_(*t*=0)=1 mM and [KB] (*t*=0)=3 mM, and in Fig. 2b, [*K* ^+^]_*o*_(*t*=0)=10 mM and [KB] (*t*=0)=10 mM. It is clear that, despite the difference in initial conditions between the two simulations, the model neuron and the glial buffering system in both simulations converge to the same steady state. The steady state is characterized by a dynamic equilibrium established between the glial buffer’s binding (*k*_*f*_) and unbinding (*k*_*b*_) of the extracellular potassium. This dynamic equilibrium is evidenced by the small but non-vanishing [*K* *B*] in the steady state. Moreover, all the steady state variables (including firing rates, [*K* ^+^]_*o*_ and [*K* *B*]) are identical between the two simulations, indicating that the steady states of the two simulations are the same. The glial buffering system enforces an upper ceiling for the extracellular potassium level in both simulations.

In Fig. 2c and d, we show the same two simulations with “pathological” glial parameters. In contrast to Fig. 2a and b, we see that the same set of different initial conditions results in distinct steady states for the two simulations. In Fig. 2c, the model neuron and the glial buffer approach a state where there is a lower [*K* ^+^]_*o*_ and a vanishing value of [*K* *B*], while in Fig. 2d, the final [*K* ^+^]_*o*_ and [*K* *B*] values are higher and the neuron is excitable with a higher firing rate. Thus, bi- or multi-stable dynamics at the single neuron level are more easily obtained with a smaller absolute value of *𝜃*, resulting into multiple fixed points (steady states) having either a lower [*K* ^+^]_*o*_ or higher [*K* ^+^]_*o*_ value.

The main factor behind this bi-stable dynamics is the parameter *𝜃*, which determines the level of bias for the forward binding rate *k*_*f*_ against the backward rate *k*_*b*_ (see equation sets (10) and (11)). With a small absolute value of *𝜃*, at a critical [*K* ^+^]_*o*_ level lower than [*K* ^+^]_*o*(*th*)_, *k*_*b*_ can be overwhelmingly larger than *k*_*f*_. The large *k*_*b*_ value rapidly lowers [*K* *B*]. Although this backward unbinding action releases *K* ^+^ back to the extracellular space, the *I*_*Kpump*_ (9) counteracts this effect and therefore [*K* ^+^]_*o*_ remains low. Moreover, the small *k*_*f*_ value (due to the bias factor *𝜃*) is not sufficient to replenish [*K* *B*] via the forward binding action. As a result, the system ends up in a state where virtually all the buffer is in the free state *B* with a low level of [*K* ^+^]_*o*_ (as in Fig. 2c).

For the neuron and glial system to exit this state, as we have shown in Fig. 2d, one can either artificially increase the values of [*K* ^+^]_*o*_ or [*K* *B*] (for example, by setting up the initial conditions). Increasing [*K* ^+^]_*o*_ to a value closer to [*K* ^+^]_*o*(*th*)_ forces *k*_*b*_ to be less biased against *k*_*f*_ (equation set (11)), so that [*K* *B*] can be more efficiently replenished and thus allows a dynamic equilibrium between [*B*] and [*K* *B*]. Increasing [*K* *B*] artificially serves similar purposes by increasing the backward unbinding release of *K* ^+^ to the extracellular space (thus increasing [*K* ^+^]_*o*_). In essence, decreasing the absolute value of *𝜃* reduces the range of [*K* ^+^]_*o*_ over which the glial buffering system can efficiently regulate, and thus makes the single neuron more susceptible to multi-stable behaviour. We show in the next section that this apparent multistability also carries over to the network simulations with abnormal glial parameters. As mentioned above, we will associate the state in which the [*K* ^+^]_*o*_ is low with the normal physiological state, and the elevated [*K* ^+^]_*o*_ level state with the “seizure” state. We also note that, although the above single-neuron analysis was based on pyramidal neurons, the same low and elevated [*K* ^+^]_*o*_ states occur in the case of FS inhibitory neurons. The effect of elevated [*K* ^+^]_*o*_ on pyramidal and FS interneuron spiking will, however, differ in the network simulations presented below.

### Sustained gamma epileptiform activity results from an abnormal but balanced glial dynamics and inhibitory synaptic strength

In this section we show how most features of gamma seizures observed in the human focal epilepsy data (Truccolo et al. 2011, 2014) can be replicated by our network model. These gamma seizures in the human data are characterized by sustained narrow band gamma LFP oscillations (∼30−60 Hz). However, few fine temporal synchrony transients exist in the spiking activities of the measured neurons. Neuronal spiking tends to be asynchronous and heterogeneous (Truccolo et al. 2011, 2014). Individual neurons also fire at a lower rate than the gamma frequency of observed LFP oscillations.

Similar neural dynamics is observed in our model simulations (Fig. 3). Critical ingredients for the appearance of sustained gamma band LFP oscillations with asynchronous neuronal firing at a lower rate include high inhibitory conductance values (for both ${g_{syn}^{{i}\rightarrow {i}}}$ and ${g_{syn}^{{i}\rightarrow {e}}}$) and an axonal conduction time delay (Brunel 2000; Brunel and Wang 2003). We also require a value of [*K* ^+^]_*o*(*th*)_ that is not too high (see equation set (11)). A moderate [*K* ^+^]_*o*(*th*)_ value ensures that the model FS inhibitory interneurons have a high excitability in the “epileptic” state, while preventing the interneurons from being overly excited to enter depolarization block. We used ${g_{syn}^{{e}\rightarrow {e}}}=0.0007$, ${g_{syn}^{{e}\rightarrow {i}}}=0.0007$, ${g_{syn}^{{i}\rightarrow {i}}}=0.025$, ${g_{syn}^{{i}\rightarrow {e}}}=0.025$, *g*_*e*0_=0.01026, *σ*_*e*_=0.0025, *g*_*i*0_=0.084, *σ*_*i*_=0.02 for this gamma seizure simulation (Table 5 introduces and defines these synaptic parameters). As for intrinsic and glial properties of model neurons, we chose *𝜃*=−0.15, [*K* ^+^]_*o*(*eq*)_=3.6, [*K* ^+^]_*o*(*th*)_=7.5, *I*_*Kmax*_=1.45, *g*_*KCa*_=5 for pyramidal cells and *𝜃*=−0.15, [*K* ^+^]_*o*(*eq*)_=3, [*K* ^+^]_*o*(*th*)_=7.5, *I*_*Kmax*_ = 1.9, *g*_*K*_ = 6.8 for interneurons. The value of *g*_*K*_ for interneurons was chosen so that they only enter depolarization block at a relatively high level of [*K* ^+^]_*o*_ ($\gtrsim 8$ mM).

Figure 3a shows the first 90 seconds of the temporal progression of several biophysical variables in the model and the time-frequency spectrum of the average membrane potentials across all the pyramidal neurons in the network (Fig. 3a, lowest panel). We use this average value as a proxy for the LFP activity.

The model cortical network during the first 40 seconds of the simulation is in the “low activity” state (in which [*K* ^+^]_*o*_ converges to ∼3mM). This “low-activity” state at the network level is similar to the single neuron level in Fig. 2c where the replenishment of [*K* *B*] is insufficient for the glial buffer to maintain a targeted level of [*K* ^+^]_*o*_. At time *t*=40s, a DC stimulation (2.5 *μ*A/cm ^2^ added to *I*_*ext*_) was applied to every neuron in the model network for a short period of 2.5 seconds. This stimulation kick-started the extracellular potassium accumulation process and forced the cortical network to enter into a “high-activity” or “epileptic” state in which the glial buffer attempts to lock into a higher [*K* ^+^]_*o*_ level (Fig. 3a, 4 ^*th*^ panel), as determined by the parameter [*K* ^+^]_*o*(*th*)_. Clear gamma band (∼40 Hz) oscillations are readily observed in the LFP power spectrum after the DC stimulation (Fig. 3a, lowest panel). Importantly, the gamma activity is sustained, even after DC stimulation is terminated, persisting until the end of the simulation.

Furthermore, at the individual neuron level, both the fast-spiking interneurons and pyramidal cells spike at lower rates of ∼ 3 and 1 spike per second, respectively, than the frequency of the gamma LFP activity (see Fig. 3a, topmost panel). The second panels from the top of Fig. 3a and b show raster plots of 2 of the 81 minicolumns in the simulation. The neuron indices in the raster plots are grouped by neuron types (pyramidal or interneuron).

It is also clear that while the LFP proxy (average membrane potential of pyramidal cells) shows gamma oscillations (Fig. 3a and b, 3 ^*rd*^ panels from top), the firing pattern of each individual neuron is not indicative of the global oscillation as observed in the LFP proxy. The density plots of the interspike intervals (ISIs) after the DC stimulation across each of the pyramidal and interneuronal populations (Fig. 3c, main plots) reveal a large variance of ISI values. Spiking in FS interneurons showed high irregularity, with the coefficient of variation (CV) of ISIs concentrating around 1 and higher values, while the CV for pyramidal neurons tended to be lower and more broadly distributed (Fig. 3c, inset plots). Irregularity of pyramidal cell spiking can be increased, nevertheless, by moderately increasing the unitary inhibition from the interneurons to the pyramidal cells. However, we observed a competition between the glial parameters (i.e. *𝜃* and [*K* ^+^]_*o*(*th*)_) and the unitary inhibitory conductance values in terms of sustaining high enough [*K* ^+^]_*o*_ values to support gamma oscillations. Increasing the unitary inhibitory conductance values affects the sustainability of gamma oscillations. Too strong ${g_{syn}^{{i}\rightarrow {e}}}$ or ${g_{syn}^{{i}\rightarrow {i}}}$ values make the gamma oscillations less sustainable, with the system immediately settling back into the original “low-activity” state. A rough mean-field estimate of the synaptic currents entering the pyramidal cells after the DC stimulation suggests that the magnitude of the inhibitory current is a few times higher than that of the excitatory current. This average inhibitory synaptic current entering a pyramidal cell can, for example, be estimated by the formula:

20 $$\begin{array}{@{}rcl@{}} \left\langle{\!I_{syn}^{i\rightarrow e}}\!\right\rangle\!&=&\!P(i\!\rightarrow\! e\!)\!\times\!{g_{syn}^{{i}\rightarrow {e}}}\!\times\! N_{i\!}\times\!\langle(\!V\!\,-\,\!E_{i}\!)\rangle\!\times\!\tau_{i}\!\times\!\langle\nu_{i}\rangle, \end{array} $$

where *ν*_*i*_ is the spike rate of the inhibitory population and *N*_*i*_ is the number of inhibitory interneurons in the network.

### Transition into spike-and-wave complex (SWC) seizures: role of synaptic inhibition and depolarization block

In this section we show how variations in synaptic inhibition and depolarization block in the examined neuronal network model can reproduce the dynamics of the observed SWC seizures. In this type of seizures, neural activity transitions into high amplitude rhythmic 2−3 Hz LFP discharges. Each SWC event in the LFP lasts for about 300 ms and is characterized by an initial fast “spike” followed by a slow wave potential. Neuronal spiking tends to occur during the LFP “spike” phase and is highly suppressed during the LFP wave phase (e.g. Truccolo et al. 2014). Preliminary examination of neuronal spiking characteristics has indicated that although both putative principal and FS inhibitory interneurons tend to increase their firing rates in the initial stages of spike-wave seizures (Ahmed et al. 2014), putative FS interneurons tend to shut down later, just before the emergence of full spike-wave discharges. Ahmed et al. (2014) observed that this termination of activity is preceded by a progressive decreasing of action potential amplitudes in FS interneurons – an indication that FS interneurons enter depolarization block, resulting eventually in the cessation of their activity.

Figure 4 shows simulation results for the case where the overall inhibitory synaptic strength is kept at a low level during the entire simulation, leading to SWC seizures. Because the aim is to have FS interneurons to eventually enter depolarization block, we set a higher [*K* ^+^]_*o*(*th*)_ and a lower *g*_*K*_ value for the interneurons than in the gamma seizure simulation (Fig. 3). Higher [*K* ^+^]_*o*(*th*)_ values increase FS interneuron excitability, while lower *g*_*K*_ values lead FS interneurons to enter depolarization block at a lower [*K* ^+^]_*o*_ level. Parameters for model neurons and glial properties of model neurons corresponded to: *𝜃*=−0.15, [*K* ^+^]_*o*(*eq*)_=3.6, [*K* ^+^]_*o*(*th*)_=8.5, *I*_*Kmax*_=1.8, *g*_*KCa*_=12 for pyramidal cells and *𝜃*=−0.15, [*K* ^+^]_*o*(*eq*)_=3, [*K* ^+^]_*o*(*th*)_=7.75, *I*_*Kmax*_=1.9, *g*_*K*_=3.5 for interneurons. Unitary inhibitory conductance values ${g_{syn}^{{i}\rightarrow {i}}}$ and ${g_{syn}^{{i}\rightarrow {e}}}$ were set here to only one-tenth of that in the gamma seizure simulation (Fig. 3). Synaptic parameters corresponded to: ${g_{syn}^{{e}\rightarrow {e}}}=0.0007$, ${g_{syn}^{{e}\rightarrow {i}}}=0.0007$, ${g_{syn}^{{i}\rightarrow {i}}}=0.0025$, ${g_{syn}^{{i}\rightarrow {e}}}=0.0025$, *g*_*e*0_=0.01016, *σ*_*e*_=0.0025, *g*_*i*0_=0.084, *σ*_*i*_=0.02.

As before, the simulation starts at a “low-activity” state (*t*=0−40 seconds) and a DC stimulation (1.6 *μ*A/cm ^2^) is delivered between *t*=40 and 42.5 seconds, “kicking” the system into a high-activity “epileptic” state. Since the overall inhibitory synaptic strength is low, but [*K* ^+^]_*o*(*th*)_ is higher than that in the gamma seizure case, FS inhibitory interneurons fire at a higher frequency after the DC stimulation than in Fig. 3, creating a positive feedback which further increases [*K* ^+^]_*o*_. When [*K* ^+^]_*o*_ reaches a critical value, inhibitory interneurons enter depolarization block, which eventually leads to cessation of inhibition (at around *t*=46 seconds in Fig. 4). We note that, in contrast to the gamma seizures, the attainment of the critical value for [*K* ^+^]_*o*_ is possible here because [*K* ^+^]_*o*(*th*)_ is set to a higher level, such that the glial potassium ceiling is higher.

After cessation of synaptic inhibition, the network dynamics is dominated by the interaction amongst pyramidal cells and evolve into rhythmic SWC discharges. Throughout, abnormal glial buffering maintains the high [*K* ^+^]_*o*_ required for keeping the interneurons in the depolarization block regime, thus creating a long-lasting “seizure” state. In the model simulations, *I*_*KCa*_ adaptation currents in the pyramidal cells and sufficiently strong excitatory-excitatory (${g_{syn}^{{e}\rightarrow {e}}}$) couplings (Van Vreeswijk and Hansel 2001; Dur-e-Ahmad et al. 2012; Nicola and Campbell 2013b; Ferguson et al. 2015) are critical for both the “spike” and “wave” firing suppression phases of the the SWC discharges.

### Transition into seizures consisting of initial low-voltage fast LFP oscillations followed by spike-and-wave discharges

Next, we demonstrate the generation of the second type of SWC seizures. In this case, the emergence of rhythmic SWC discharges is preceded by low voltage higher frequency (∼10−20 Hz) LFP oscillations. Neuronal spiking activity during these fast oscillations tends to be asynchronous as in the previously examined gamma seizures. In other words, while there is a clear fast frequency oscillation at the LFP level, individual neurons spike at a lower rate without obvious synchrony with the global (i.e. LFP) oscillation patterns. The similarity of the firing dynamics of individual neurons between these transient oscillations and gamma seizures leads us to speculate that both phenomena might share a common synaptic and glial mechanism. As shown in Fig. 5, the seizure begins with low voltage fast (∼20 Hz) LFP oscillations which eventually evolve into low-frequency rhythmic SWC discharges. Similarly to the gamma seizures (Fig. 3), the initial fast oscillations in the model require a high overall inhibitory synaptic strength, while the evolution and maintenance of spike-wave discharges require the opposite.

To reproduce this type of seizure dynamics, the following sequence of events was implemented in the model: a substantial increase in inhibitory synaptic strength at seizure transition (leading to low voltage fast oscillations) is followed by a rapid decrease, which is hypothesized here to occur via GABA depletion (Zhang et al. 2012). Once synaptic inhibition is below normal levels, the same path as in the first type of SWC seizures follows: inhibitory interneurons enter depolarization block and the network evolves into rhythmic SWC discharges supported mostly by the population of pyramidal neurons. Specifically, the synaptic parameters ${g_{syn}^{{i}\rightarrow {i}}}$ and ${g_{syn}^{{i}\rightarrow {e}}}$ were set to constant values during the entire simulation.

To increase the effective inhibitory synaptic strength during seizure transition (i.e. during the DC stimulation phase, *t*=40−42.5s), we let the maximal value of the gating variable per spike, *s*_*max*_, for interneurons (equation set (18)), vary temporally instead of being a constant as in Figs. 3 and 4. The value of *s*_*max*_ for interneurons was set to 1 at the beginning of the simulation (Table 5) and kept at this value during the “low activity” phase (*t*=0−40s). After that, *s*_*max*_ for interneurons asymptotically approached a terminal value $s_{max}^{term}$ during the DC stimulation phase according to the following formula:

21 $$\begin{array}{@{}rcl@{}} {s_{max}}(t+\delta t)&=&{s_{max}}(t)\times\left( 1+\left( \frac{{s_{max}^{term}}}{{s_{max}}(t)}-1\right)\times\frac{\delta t}{\tau_{rise}}\right), \end{array} $$

where *τ*_*rise*_=62.5ms is the time constant of the increase in *s*_*max*_ and ${s_{max}^{term}} = 70$ is the asymptotic terminal value of *s*_*max*_ (i.e. 70 times the value of original *s*_*max*_ before the DC stimulation phase).

The increased *s*_*max*_ value for interneurons was then held constant during the fast oscillations phase (from 42.5 to 49.5 seconds). After that it was decreased to simulate the loss of inhibition. We set *s*_*max*_→0.1×*s*_*max*_ for individual interneuron after each spike of the same interneuron during this “depletion” phase. (The start of this depletion phase is marked by a red triangle in Fig. 5.) Eventually the inhibition approaches zero, the “depleted” state.

In summary, Fig. 5 shows the emergence of clear low voltage fast (∼ 20 Hz) LFP oscillations right after the onset of the DC stimulation. These fast oscillations disappear shortly after the start of the inhibition “depletion” phase (marked by a red triangle in Fig. 5). The strong build up of inhibitory synaptic strength (up to 70 times the initial strength) during the DC stimulation is necessary because it slows the rise of [*K* ^+^]_*o*_ during DC stimulation and keeps the interneurons from entering depolarization block. Moreover, this very high level of synaptic inhibition keeps the population frequency in the ∼10−20 Hz range. A lower, but still large inhibitory strength would have led to the emergence of gamma oscillations, as in Fig. 3.

The initial decrease in [*K* ^+^]_*o*_ during the transient fast oscillations (Fig. 5a, fourth plot from top) is due to the strong inhibition which limits the firing of inhibitory interneurons. As inhibition begins to wear off at *t*=49.5 seconds, the trajectory of [*K* ^+^]_*o*_ reverses its course because both pyramidal cells and interneurons fire more as a result of decreased inhibitory synaptic strength. Finally, when [*K* ^+^]_*o*_ reaches a sufficiently high level (∼7 mM for this simulation), the interneurons enter depolarization block and inhibition ceases, as clearly shown by the complete termination of interneuron firing (Fig. 5b, raster plot at around *t*=63 seconds). After cessation of inhibition, SWC discharges emerge supported only by the activity of interacting pyramidal neurons. With the exception of the time varying nature of *s*_max_ for interneurons and a DC stimulation here set to 2.5 instead of 1.6 *μ*A/cm^2^, the numerical values of the synaptic (including ${g_{syn}^{{i}\rightarrow {i}}}$ and ${g_{syn}^{{i}\rightarrow {e}}}$), intrinsic and glial parameters were the same as those for the first type of SWC seizures (Fig. 4).

## Discussion

Our study is motivated by recent microelectrode array recordings of ensembles of single neurons and high-density LFP activity in human neocortex during propagated focal seizures (Truccolo et al. 2011; Schevon et al. 2012; Ahmed et al. 2014; Truccolo et al. 2014; Wagner et al. 2015). These recordings suggest different ways via which seizures starting in a focal site can recruit more distal neocortical areas. We used a biophysical cortical network model consisting of conductance-based neurons, coupled with glial buffer for [*K* ^+^]_*o*_, to examine potential mechanisms underlying transitions into ictal states in recruited neocortical areas during seizure spread. Through the analyses and simulations of single neuron and network models, we have demonstrated that neocortical networks can be made susceptible to seizures as a result of abnormal glial potassium buffering and changes in synaptic inhibition.

First, at the single neuron level, the imbalance in the conversion between potassium buffer in the bound and unbound forms leads to bi-stability such that one state has a lower [*K* ^+^]_*o*_ level (thus lower excitability) than the other. This bi-stability also carries over to the network level. Perturbation either by noise or by DC stimulation can “kick-start” the high [*K* ^+^]_*o*_ hyper-excitable state from the low [*K* ^+^]_*o*_ state. Second, we then showed that the three major types of seizure transitions as observed in the human microelectrode array recordings can be reproduced when these pathological glial potassium dynamics interact with various synaptic and intrinsic parameter settings for inhibitory interneurons.

In our simulations, transitions into gamma (∼30−60 Hz) ictal activity were obtained with a high inhibitory conductance between cortical neurons. Such high inhibitory synaptic strength was required to prevent the interneurons from entering depolarization block during the seizure state. Moreover, since high inhibitory strength tends to decrease [*K* ^+^]_*o*_, the long-lasting gamma seizure state was the result of a subtle balance between high inhibitory conductance values and the accumulation of [*K* ^+^]_*o*_ due to abnormal glial potassium buffering dynamics.

For transitions into SWC ictal activity, the cortical network can either experience a generally low value of inhibitory conductance between cortical neurons, or an initial high inhibitory synaptic strength followed by inhibition breakdown. In the first scenario, the transition into SWC was direct and involved interneurons entering depolarization block because of the low inhibitory synaptic strength. As soon as the interneurons entered depolarization block, inhibition ceased. At that point, neuronal spiking activity supported only by interacting pyramidal cells led to recurring SWC discharges. In the second SWC scenario, there was a buildup of inhibitory synaptic strength prior to the transition into spike-wave discharges. The result of this inhibition buildup is the occurrence of transient low-amplitude fast oscillations around 10−20 Hz. This buildup of inhibitory synaptic strength was followed by a wearing off of inhibition (e.g. through GABA depletion), thus providing a low inhibition environment for interneurons to enter depolarization block and the population of pyramidal cells to eventually sustain recurring SWC discharges.

### Abnormal glial buffering and its relationship to seizure

In this work we have established a computational model of seizure through the occurrence of bi-stability at the single neuron level. This bi-stability is the result of a hypothesized pathological glial potassium dynamics in which there is an imbalance between potassium buffers in their bound and unbound forms. Bi- (or multi)stability, either at the single neuron or at the network level, has been implicated in numerous physiological and pathological conditions in the brain (Van Ooyen et al. 1992; Sasaki et al. 2007; Freyer et al. 2009; Fröhlich et al. 2010; Anderson et al. 2012; Ho et al. 2012; He 2014; Ho et al. 2014; Hübel et al. 2014). In the particular case of epileptic seizures, Fröhlich et al. (2010) have demonstrated in computational models that it is possible for network bistability to exist even when the glial potassium dynamics are within a physiological range (through a higher absolute value of *𝜃*, the parameter determining the bias favouring forward versus backward binding rate). In this scenario, Fröhlich et al. (2010) associate higher [*K* ^+^]_*o*_ states with epileptic seizures. In the models used here, we did not observe bi-stability either at the single neuron or network level when *𝜃* was set to physiological values. We only observed bi-stability with pathological (i.e. smaller in absolute value) values of *𝜃*. We have singled out (via single neuron simulations) the differential in transition rates between [*B*] and [*K* *B*] as the important factor underlying such bi-stability. The fact that Fröhlich et al. (2010) used two-compartment neuron models, while we used single-compartment, might explain this difference between the two studies. Moreover, different synaptic connection parameters were used by Fröhlich et al. (2010). Different synaptic connectivity might affect the length and strength of perturbation required to elicit the bi-stable behaviour, if at all possible. A more thorough examination of how glial potassium buffering may affect neuronal dynamics should involve detailed bifurcation analyses of the combined neuron-glial system (Touboul 2008; Nicola and Campbell 2013a; Kim and Nykamp 2014; Nicola and Campbell 2014). Such analyses could determine the intrinsic and glial parameters that allow multistability. From the existence and stability of these states, one would expect to gain some insight into how resilient the network is to seizure initiation and propagation. Although we have focused on their role on seizure propagation, potassium and glial buffering may also play an important role in seizure termination (Kramer et al. 2012; González-Ramírez et al. 2015).

### Changes in inhibitory and excitatory conductances

Our focus on changes in inhibition was motivated by several previous (*in vivo/vitro*) animal models (Trevelyan et al. 2006; Zhang et al. 2012; Grasse et al. 2013; žiburkus et al. 2013; Uva et al. 2015) and *in vitro* studies of human epileptic cortical tissue (e.g. D’Antuono et al. 2004), where inhibitory interneuron activity appears to change significantly during preictal and initial periods of the seizure, before major changes in principal cells are detected. Recent work by žiburkus et al. (2013) has also examined in detail how changes in both inhibitory and excitatory conductances may lead to imbalances in excitation/inhibition preceding and during seizure like events in hippocampal slices under the potassium channel blocker 4-aminopyridine and reduced extracellular magnesium. In that respect, we emphasize that the changes in inhibition as implemented in our simulations did not only affect inhibitory conductances. Changes in inhibition in our model were implemented in part by systematically varying the levels of unitary synaptic inhibition, which in turn affected several other network properties such as the number of neurons generating spikes at any given time, and thus firing rates in both populations of pyramidal neurons and FS interneurons. In this way, by varying unitary synaptic inhibition, the E-I balance in the simulated networks also varied dynamically, with both excitatory and inhibitory conductances being altered. In addition, after transitions into spike-wave discharges in the simulated SWC seizures (Figs. 4 and 5), these discharges were supported only by the synaptic interactions among pyramidal neurons.

Furthermore, we note that there are a couple of important differences between the animal model in žiburkus et al. (2006, 2013) and our model simulations. First, apart from temporary depolarization block periods, the activity of inhibitory neurons remained throughout the seizures (i.e. until seizure termination) in žiburkus et al.’s model. In our computational model, it is only during gamma seizures that the spiking activity of FS inhibitory interneurons is preserved throughout the seizure and depolarization block is absent. By contrast, FS interneurons enter depolarization block and remain in that state during the SWC seizures. Second, while in žiburkus et al.’s study transient depolarization block events in pyramidal neurons are also present and play an important role, in our computational model pyramidal neurons tend to be robust to depolarization block both in SWC and gamma seizures. This robustness resulted primarily from the slow calcium-mediated potassium afterhyperpolarization (AHP) currents (*I*_*KCa*_, Eq. (7)) in the model pyramidal neurons, which prevented them from evolving into regimes of high enough firing rates required for depolarization block. These features of the biophysical model examined here seem consistent with the dynamics of propagated seizures (in neocortical patches distal from putative onset areas) observed in our human data. First, both inhibitory and excitatory spiking activity appears to be preserved throughout the gamma seizures (Truccolo et al. 2011). Second, in SWC seizures, spiking in putative FS interneurons appears to terminate before the transition into large amplitude spike wave discharges. This cessation of FS interneuron spiking activity is accompanied by signatures of depolarization block, while no similar signatures were found in putative principal cells (Ahmed et al. 2014).

### Network inhibition and its relationship to seizure propagation

Using biophysical neuronal network models of neocortical patches, we have demonstrated how FS inhibitory interneurons may play an important role in three types of observed human seizures. In particular, we have quantified the role of FS interneurons by varying the inhibitory synaptic strength and controlling how easily interneurons enter depolarization block. For the two types of SWC seizures modeled here (Figs. 4 and 5), ultimately it is the failure of inhibition (when the FS inhibitory interneurons enter depolarization block) that leads the network to transition into SWC activity. We examined two main scenarios that led to the failure of inhibition, resulting in SWC seizures. In the first scenario, FS inhibitory interneurons enter depolarization block when there is an overall low inhibitory synaptic strength. When interneurons are excited during seizure initiation, the lower inhibitory synaptic strength allows interneurons to fire at a higher frequency, thus hastening the [*K* ^+^]_*o*_ accumulation process and leading eventually to depolarization block (Fig. 4). In the second scenario, an initially large inhibitory synaptic strength is followed by GABA depletion (Zhang et al. 2012). Initially, interneurons are mutually inhibited by the large inhibitory synaptic strength and their firing frequencies are too low to trigger [*K* ^+^]_*o*_ accumulation. If such level of inhibition was not perturbed, seizure propagation would fail. The decreased effective inhibitory strength resulting from subsequent GABA depletion allows for higher firing rates in interneurons (and principal cells) to develop, again leading eventually to depolarization block in inhibitory interneurons (Fig. 5).

Some previous studies have emphasized the role of feedforward inhibition in seizure propagation in neocortex (Trevelyan et al. 2006; Schevon et al. 2012; for a review see Paz and Huguenard 2015). The main idea is that the propagation of initially localized ictal activity to more distal cortical sites involves predominantly feedforward inhibition. This feedforward inhibitory drive could be a mechanism for the initial strengthening of inhibition during seizure propagation assumed in our model in two of the three seizure propagation scenarios (gamma and SWC with preceding fast oscillations). Furthermore, Trevelyan and colleagues (Trevelyan et al. 2006; Schevon et al. 2012) have emphasized the role of an “inhibitory restraint” or “veto” mechanism during neocortical propagation of focal seizures. Inhibitory veto of seizure propagation has been shown in low Mg ^2+^ mouse models of epilepsy (Trevelyan et al. 2006) and has been argued to be present also in human focal seizures (Schevon et al. 2012). This inhibitory veto mechanism would lead to “penumbra areas” ahead of the ictal wavefront. The failure of such inhibitory veto would allow the successful propagation of the ictal wavefront into new recruited ictal areas. Otherwise, the inhibition would be strong enough to contain the seizure spread. In this way, one could also argue that the gamma seizures examined here would represent a failure in seizure propagation. This possibility remains an open and important experimental question. Given that these sustained gamma oscillations reflect abnormal dynamics during secondarily generalized focal seizures, we currently consider this activity as propagated seizures. The gamma seizures in our model (Fig. 3) represent a situation in which there is a balance between enhanced inhibition and [*K* ^+^]_*o*_ accumulation. In other words, the seizure propagates while inhibitory interneuronal activity is preserved. In a related experimental study, our group (Lu et al. 2015) has shown that constant, strong enough depolarization driven by optogenetic stimulation can induce sustained (∼50 Hz) gamma oscillations in healthy primate motor cortex.

In view of the strong inhibition in simulations of gamma seizures (Fig. 3), we do not preclude the possibility that some of the “high-activity” states in the simulated gamma seizures may in fact be long-lasting transients as opposed to asymptotic stable states. In other words, the elevated [*K* ^+^]_*o*_ in the “high-activity” states may nevertheless settle back to a much lower value after a long enough time. However, this issue has little effect on our main claims in this paper, given the long time scale (at least in the order of minutes) over which [*K* ^+^]_*o*_ remains elevated and increasing.

### Model limitations and future work

Our analyses and simulations show how different types of seizure transitions observed in intracortical recordings of propagated human focal seizures can be accounted for by variations in synaptic inhibition and extracellular potassium concentration. Several limitations of the model, however, warrant future improvement. First, although the interaction of [*K* ^+^]_*o*_ and inhibitory synaptic dynamics can account for the phenomena observed in our human epilepsy data (Ahmed et al. 2014), several other ionic or synaptic mechanisms are possible and have been studied in different animal models of epilepsy (e.g. Alfonsa et al. 2015).

Second, our model exploration was restricted to the transition into ictal states. In particular, we did not examine how seizures terminate, which is also a complex topic and may involve multiple ionic and synaptic components (such as glutamate depletion–Lado and Moshé 2008; GABA upregulation–Wen et al. 2015; fluctuations in ionic concentrations–Krishnan and Bazhenov 2011; Kramer et al. 2012). A more detailed model, including more complex ionic dynamics, will be required to address this issue. Furthermore, gap junction effects were not included in the model. Although electrical synapses have been suggested as an underlying mechanism for interneuronal bursting (Skinner et al. 1999) and synchronous population gamma oscillations (Traub et al. 2001), neither of these two features appeared to be prominent in our human data where neuronal spiking during gamma seizures is largely asynchronous. Thus, it remains an open question whether gap junction effects are critical to account for transitions to and maintenance of gamma seizures. Nevertheless, gap junctions may still play an important role during SWC discharges as previously hypothesized by Traub and colleagues (e.g. Traub et al. 2005). We hope to address these open issues in the future.

Third, in the hypothesized contributing factors to seizure transitions examined here, we have remained agnostic about what ultimately leads to transient dysfunctions in inhibitory activity and in potassium glial-buffering processes. We note that several other factors involving changes in oxygen, Na+/K+ ATP pumps and cell volume (e.g. Ingram et al. 2014; Wei et al. 2014a), for example, can also contribute to elevations in extracellular potassium. We also emphasize that the goal in this study is to replicate the dynamics observed in propagated seizures, in other words, ictal dynamics in neocortical patches distal to the putative seizure onset zones. As described in Truccolo et al. (2011, 2014) and Wagner et al. (2015), our recorded neocortical patches were close, but distal to the putative seizure onset zones. The dynamics involving seizure initiation in seizure onset/focus areas might involve different mechanisms.

As stated above, we have focused on fast time-scale changes in synaptic inhibition which have been reported in several previous animal studies and *in vitro* studies of human epileptic cortical tissue. Although these studies have provided some initial evidence for transient changes in inhibitory interneuron activity preceding seizures, it remains a major open question how these dysfunctions in interneuron network activity (and potassium glial buffering) would arise in the longer time scale of epileptogenesis.
